# Pharmacological Reversal of Histone Methylation Presensitizes Pancreatic Cancer Cells to Nucleoside Drugs: *In Vitro* Optimization and Novel Nanoparticle Delivery Studies

**DOI:** 10.1371/journal.pone.0071196

**Published:** 2013-08-06

**Authors:** Sau Wai Hung, Hardik Mody, Sean Marrache, Yangzom D. Bhutia, Franklin Davis, Jong Hyun Cho, Jason Zastre, Shanta Dhar, Chung K. Chu, Rajgopal Govindarajan

**Affiliations:** 1 Department of Pharmaceutical and Biomedical Sciences, The University of Georgia, Athens, Georgia, United States of America; 2 Department of Chemistry, The University of Georgia, Athens, Georgia, United States of America; 3 Department of Biological Sciences, The University of Georgia, Athens, Georgia, United States of America; Enzo Life Sciences, Inc., United States of America

## Abstract

We evaluated the potential of an investigational histone methylation reversal agent, 3-deazaneplanocin A (DZNep), in improving the chemosensitivity of pancreatic cancer to nucleoside analogs (i.e., gemcitabine). DZNep brought delayed but selective cytotoxicity to pancreatic cancer cells without affecting normal human pancreatic ductal epithelial (HPDE) cells. Co-exposure of DZNep and gemcitabine induced cytotoxic additivity or synergism in both well- and poorly-differentiated pancreatic cell lines by increased apoptosis. In contrast, DZNep exerted antagonism with gemcitabine against HPDE cells with significant reduction in cytotoxicity compared with the gemcitabine-alone regimen. DZNep marginally depended on purine nucleoside transporters for its cytotoxicity, but the transport dependence was circumvented by acyl derivatization. Drug exposure studies revealed that a short priming with DZNep followed by gemcitabine treatment rather than co-treatment of both agents to produce a maximal chemosensitization response in both gemcitabine-sensitive and gemcitabine-resistant pancreatic cancer cells. DZNep rapidly and reversibly decreased trimethylation of histone H3 lysine 27 but increased trimethylation of lysine 9 in an EZH2- and JMJD1A/2C-dependent manner, respectively. However, DZNep potentiation of nucleoside analog chemosensitization was found to be temporally coupled to trimethylation changes in lysine 27 and not lysine 9. Polymeric nanoparticles engineered to chronologically release DZNep followed by gemcitabine produced pronounced chemosensitization and dose-lowering effects. Together, our results identify that an optimized DZNep exposure can presensitize pancreatic cancer cells to anticancer nucleoside analogs through the reversal of histone methylation, emphasizing the promising clinical utilities of epigenetic reversal agents in future pancreatic cancer combination therapies.

## Introduction

Polycomb group proteins (PcGs) can remodel chromatin by influencing the degree of compaction, leading to epigenetic gene silencing. Polycomb Repressive Complex 2 (PRC2), one of the two classes of PcGs, induces histone methyltransferase activity primarily by trimethylating histone H3 at lysine 27 (H3K27me3), mediating silencing of tumor suppressor genes. The catalytic subunit of PRC2 is Enhancer of Zeste Homolog 2 (EZH2), in which the SET domain constitutes the active site for histone H3K27 methylation [1]. Studies support EZH2 as a key player in the development and progression of tumors due to its ability to alter gene expressions including those involved in cell cycle control, cell migration, and DNA repair [2]. EZH2 is crucial in the chromatin control of genetic reprogramming of cancer stem cell self-renewal and differentiation that have been implicated in chemoresistance [3]–[6].

As a marker of advanced and metastatic disease in many solid tumors, EZH2 overexpression has been reported in pancreatic cancers, particularly those that are poorly differentiated [6], [7]. EZH2 was found to be upregulated by oncogenic RAS through MEK-ERK signaling, leading to the downregulation of tumor suppressors such as RUNX3 and p27(Kip1) [6], [8], [9]. EZH2 depletion led to cell cycle arrest at the G1/S transition, suggesting the protein may repress the tumor suppressing p27 gene [10]. Similarly, knockdown of EZH2 resulted in a significant decrease in cellular proliferation and invasiveness [6], [7], [11] and sensitized pancreatic cancer cells to doxorubicin and gemcitabine, revealing the potential of an EZH2 inhibitor-chemotherapeutic combination therapy [6]. *In vivo*, suppressing EZH2 diminished tumorigenicity and inhibited pancreatic cancer metastasis [7]. Clinically, positive correlations have been observed between EZH2 expression and advanced pancreatic cancer stage and grade in patients [11]. In many cases, high levels of EZH2 in cancer were also significantly associated with decreased E-cadherin expression and highly aggressive disease. In gemcitabine-treated patients, significantly longer survival was observed in patients with low rather than high EZH2 expression [12]. Consequently, EZH2 may be a significant prognostic value for overall survival in pancreatic cancer patients [13].

Recently, it has been shown that a potent chemical inhibitor of S-adenosylhomocysteine hydrolase, 3-deazaneplanocin A (DZNep), modulates chromatin through indirect (i.e., reducing methyl group availability) inhibition of histone methyltransferases including EZH2 [14], [15]. DZNep, a carbocyclic analog of adenosine, depletes cellular levels of the PRC2 components while inhibiting the associated H3K27me3 [16]. While the mechanisms and effects of DZNep have been studied in numerous solid tumors and leukemia [2], [3], [14], [15], [17]–[21], less is known about the potential of this compound for pancreatic cancer treatment. Nevertheless, its current potential for reducing EZH2 levels, reverting epithelial-to-mesenchymal transition (EMT), and preventing tumor progression, makes it a highly promising antimetastatic agent [5]. The therapeutic potential of DZNep in combination with other agents, such as polyphenols and histone deacetylase inhibitors, has begun to emerge with encouraging results [14], [15]. Since increasing evidence suggests that future cancer therapies will take advantage of the synergistic effects achieved from different combinations of epigenetic reversal and conventional antitumor agents [22], we investigated the potential of the DZNep-gemcitabine combination for improving anticancer activity in pancreatic cancer. Our results identified that histone methylation reversal by DZNep presensitizes pancreatic cancer cells to gemcitabine. Optimization of the drug combination through dosage and delivery methods was further conducted.

## Materials and Methods

### Reagents

Radiolabeled (^3^H) gemcitabine, adenosine, thymidine, and guanosine were obtained from Moravek Biochemicals and Radiochemicals (Brea, CA), while cold gemcitabine was from ChemieTek (Indianapolis, IN). Adenosine and guanosine were kindly provided by Dr. Chung K. Chu (University of Georgia). Thymidine, uridine, nitrobenzyl mercaptopurine riboside (NBMPR), and 3-(4,5-dimethylthiazol-2-yl)-2,5-diphenyltetrazolium bromide (MTT) were obtained from Sigma-Aldrich (St. Louis, MO). Dimethylsulfoxide (DMSO) was purchased from Macron Chemicals (Center Valley, PA), and the bicinchoninic acid (BCA) protein assay reagent was from Thermo Scientific Pierce (Rockford, IL). Plastic wares for cell culture were obtained from Corning (Corning, NY).

### Cell Culture

The pancreatic cancer cell lines (AsPC-1, BxPC-3, Capan-1, MIA PaCa- 2, and PANC-1) and MCF-10A cells were received from the American Type Culture Collection (ATCC; Manassas, VA) cell bank. These cell lines were propagated, expanded, and frozen immediately after arrival. The cells revived from the frozen stock were used within 10–20 passages, not exceeding a period of 2–3 months. The ATCC uses morphological, cytogenetic and DNA profile analysis for characterization of cell lines. Human pancreatic ductal epithelial (HPDE) cells [23] were kindly received from Dr. Ming Tsao of the Ontario Cancer Institute (Toronto, Canada). The L3.6pl cell line [24] was kindly received from Dr. Isiah D. Fidler at The University of Texas MD Anderson Cancer Center (Houston, TX). The HPDE and L3.6pl cell lines were handled as other cell lines and were genotyped by DNA fingerprinting (PowerPlex 16, Promega, Inc.) as per the manufacturer’s instructions. The 293T cell line was kindly received from Dr. J. Michael Thomson of The University of Georgia (Athens, GA). The growth conditions of cell lines were performed as described previously [25].

### MTT Cytotoxicity Assay

Cells were seeded at a density of 3×10^3^ cells/well in a 96-well microtiter plate and grown to 90–95% confluency. After treatment, 50 µl of MTT solution (5 mg/ml in PBS) were added to each well, and the plates were incubated for 2 h. MTT formazan crystals were dissolved in 100 µl/well of DMSO by shaking the plates on a rocking platform. A 96-well scanner was used to measure the spectrophotometric absorbance at 490 nm. The absorbance at 650 nm was used for background subtraction. The 50% inhibitory concentration (IC_50_) was determined using GraphPad Prism 5.0 software.

### Caspase 3 Assay

Apoptosis was measured using the Fluorimetric Caspase 3 Assay Kit from Sigma-Aldrich (Cat. No. CASP3F) as per the manufacturer’s instructions. Briefly, 10^4^ cells/well in a 96-well plate were treated with DZNep and/or gemcitabine for 72 h. Cells were then lysed and incubated on ice for 15–20 min. After adding assay buffer containing the Ac-DEVD-AMC substrate, samples were transferred to a black 96-well plate, and fluorescence was read every 10 min for 1 h at room temperature (360 nm excitation, 460 nm emission). The appropriate blank (reaction mixture), positive control (caspase 3), and negative control (caspase 3+ caspase 3 inhibitor) were also conducted.

.

### Drug Interaction Studies

The combination index plots for DZNep and gemcitabine were estimated using the method developed by Chou and Talalay and the CalcuSyn software [26].

### Nucleoside Uptake in Cells and *Xenopus* Oocytes

These procedures were performed as previously described [27], [28].

### Generation of *Xenopus* Oocyte Expression Constructs

The full-length IMAGE cDNA clones of the transporters (hENT1: clone ID 3010092, accession BC008954; hENT2: clone ID 9051840, accession BC143335; hCNT1: clone ID 8991920, accession BC 126204; hCNT3: clone ID 7939668, accession BC093823) were obtained from Open Biosystems (Huntsville, AL). Subcloning of the genes into the *Xenopus* oocyte expression vector, pOX, was completed using the primer sets designated in Table 1.

**Table 1:** Restriction sites and sequences of primers used for cloning.

Gene	Forward		Reverse	
	Restriction Site	Sequence	Restriction Site	Sequence
pOX-hENT1	SalI	5′-GCACGTCGACCAATAATGACAACCAGTCAC-3′	XbaI	5′-CGTGTTCTAGATCACACAATTGCCCG-3′
pOX-hENT2	SalI	5′-GATATAGTCGACCAATAATGGCGCGAGGAGAC-3′	XbaI	5′-CTATCTCTAGATCAGAGCAGCGCCTTGAAGAG-3′
pOX-hCNT2	HindIII	5′-GCCCGAAGCTTCAATAATGGAGAAAGCAAGTGG-3′	XbaI	5′-CGTCGTCTAGATTAGGCACAGACGGTATTG-3′
pOX-hCNT3	HindIII	5′-CGCACAAGCTTCAATAATGGAGCTGAGGAG-3′	SpeI	5′-CCGCGCACTAGTTCAAAATGTATTAGAGATCCC-3′

### Western Blotting

Western blotting was conducted as previously described [25]. The rabbit polyclonal anti-histone H3K4TM, H3K9MM, H3K9DM, H3K27MM, H3K27DM, H4K20DM, and H4K20TM were from Millipore (Billerica, MA), as well as the rabbit polyclonal anti-EED antibody. Also obtained from Millipore were the mouse monoclonal anti-histone H3K9TM, H3K27TM, and H4K20MM antibodies, as well as the mouse monoclonal anti-EZH2 (clone BD43) and anti-SUZ12 (clone 3C1.2) antibodies. The rabbit polyclonal anti-JMJD1A and anti-JMJD2C antibodies were acquired from Sigma-Aldrich. The mouse monoclonal anti-β-actin antibody was also purchased from Sigma-Alrich, and the HRP-conjugated secondary antibodies were from Bethyl Laboratories (Montgomery, TX).

### Synthesis of Parent Nucleosides and Derivatives

Troxacitabine and its lipophilic prodrug, as previously described (compound 2K [29]; compound 6h [30]; C_24_H_41_N_3_O_5_), were obtained from Dr. Chung K. Chu (University of Georgia). The synthesis of a DZNep analog began from D-ribose. D-ribose was treated with 2,2-dimethoxypropane in the presence of a catalytic amount of *p*-toluenesulfonic acid to give a isopropylidine derivative, followed by the protection of the primary alcohol with triphenylmethyl chloride. The protected lactol was then reacted with vinyl magnesium bromide to give a single diastereomericdiol, which was subsequently protected with TBDMS only at the allylic hydroxyl position to afford silyldienol. To incorporate another double bond for the ring-closing metathesis reaction, the protected secondary alcohol was oxidized to a ketone by Swern oxidation, followed by a Wittig reaction with methyltriphenylphosphonium bromide and n-BuLi to provide the diene. The silyl group from the diene was removed with TBAF to give a less sterically demanding dienol, which was then converted to cyclopentenol with a second-generation Grubbs catalyst in good yield. In order to carry out the Mitsunobu coupling, bis-Boc-3-deaza-adenine was prepared. Mitsunobu coupling provided the desired N-9 isomer as a major product. Removal of protection groups using 2N-HCl in methanol produced DZNep. Three alcohol groups of DZNep were protected with TBS which was substituted to the two amide prodrugs (carbon chains of C6 to C8) by acylation of the N^6^-NH_2_ group. The compound was finally fully deprotected by TBAF to give the DZNep prodrug 1 (C_20_H_29_ClN_4_O_4_) and DZNep Prodrug 2 (C_18_H_24_N_4_O_4_). The syntheses procedures and physicochemical characterizations of the compounds will be published elsewhere.

### Nanoparticle Formulation

All chemicals were purchased from Sigma Aldrich and used without further purification unless otherwise noted. PLGA-COOH with an intrinsic viscosity of 0.18 dL/g was purchased from Lactel. The (5-carboxypentyl)triphenylphosphonium (TPP) cation was synthesized according to literature methods [31]. HO-PEG-OH with a MW of 3,350 g/mol was purchased from Sigma Aldrich. DSPE-PEG-COOH with a MW of 2,000 g/mol was purchased from Avanti Polar Lipids. Polyvinyl alcohol (PVA) with an average molecular weight of 10,000–26,000 was purchased from Alfa Aesar. Distilled water was purified by passage through a Millipore Milli-Q Biocel water purification system (18.2 MΩ) with a 0.22 µm filter. HPLC analyses were carried out using an Agilent 1200 series instrument. Size and zeta potential measurements were carried out on a Malvern Zetasizer Nanoseries instrument. Gel permeation chromatography (GPC) data was collected on a Shimadzu LC20-AD Prominence liquid chromatographer. All NMR spectra were recorded on a Varian 400 MHz NMR. TEM images were taken on a Tecnai 20 FEM microscope. Ultrasonication was performed on a Misonix S-4000 Ultrasonic liquid processor.

### Synthesis of PLGA-*b*-PEG-OH

Synthesis of PLGA-PEG-*b*-OH was performed as previously described [32].

### Synthesis of PLGA-*b*-PEG-TPP

Synthesis of PLGA-PEG-*b*-TPP was performed as previously described [32].

### Synthesis of Gemcitabine Encapsulated Nanoparticles (Gem-PLGA-*b*-PEG-OH-NPs)

Gemcitabine (10 mg/mL in nanopure H_2_O) was emulsified with PLGA-*b*-PEG-OH (5 mg in CH_2_Cl_2_) using probe sonication for one minute (Amplitude: 40% of 600 W power, Pulse: 1 sec on, 1 sec off). The primary emulsion was emulsified again by adding the water-in-oil emulsion to 2 mL of nanopure water containing 0.5% polyby 8 mL of nanopure water containing 0.05% PVA and sonicated using the conditions mentioned above. Organic solvent was removed by washing three times using an Amicon filtration membrane with a 100 kDa cut-off. The NPs were resuspended in nanopure water and stored at 4°C until further use. Dynamic light scattering (DLS) measurements were carried to determine size, polydispersity index (PDI), and zeta potential of the NPs. NPs were characterized using TEM at an acceleration voltage of 200 kV. The TEM samples were prepared by depositing 8 µL of the NPs (5 mg/mL) onto a 200-mesh carbon-coated copper grid. Samples were blotted away after 15 min and grids were negatively stained with sterile 2% (w/v) uranyl acetate aqueous solution for 15 min.

### Synthesis of DZNep Encapsulated Nanoparticles (DZNep-PLGA-*b*-PEG-OH-NPs)

DZNep (10 mg/mL in nanopure H_2_O) was emulsified with PLGA-*b*-PEG-OH (5 mg in CH_2_Cl_2_) using probe sonication for one minute (Amplitude 40% of 600 W power, Pulse: 1 sec on, 1 sec off). The primary emulsion was emulsified again by adding the above formed water-in-oil emulsion to 2 mL of nanopure water containing 0.5% PVA and sonicated using similar conditions. Finally, this emulsion was re-emulsified using 8 mL of nanopure water containing 0.05% PVA under sonication using the similar conditions as before. Organic solvent was removed by washing three times using an Amicon filtration membrane with a 100 kDa cut-off. The NPs were resuspended in nanopure water and stored at 4°C until further use. DLS measurements were carried to determine size, polydispersity index (PDI), and zeta potential of the NPs. NPs were characterized using TEM as mentioned above.

### Synthesis of Gemcitabine and DZNep Encapsulated NPs (Gem-DZNep-PLGA-*b*-PEG-OH-NPs)

Gemcitabine and DZNep co-encapsulated PLGA-b-PEG-OH NPs were following the exact procedure mentioned above except the first step where DZNep (10 mg/mL in nanopure H_2_O) and Gemcitabine (10 mg/mL in nanopure H_2_O) was emulsified with PLGA-PEG-OH (5 mg in CH_2_Cl_2_). Organic solvent was removed by washing three times using an Amicon filtration membrane with a 100 kDa cut-off. The NPs were resuspended in nanopure water and stored at 4°C until further use. DLS measurements were carried to determine size, polydispersity index (PDI), and zeta potential of the NPs. NPs were characterized using TEM as mentioned above.

### Synthesis of Controlled Release Gem-DZNep-PLGA-*b*-PEG-TPP-NPs

Gemcitabine (10 mg/mL in nanopure H_2_O) was emulsified with PLGA-*b*-PEG-TPP (5 mg in CH_2_Cl_2_) using probe sonication for one minute (Amplitude: 40% of 600 W power, Pulse: 1 sec on, 1 sec off). The primary emulsion was emulsified again by adding the water-in-oil emulsion to 2 mL of nanopure water containing 0.5% PVA and sonicated using similar conditions. Finally this emulsion was emulsified using 8 mL of nanopure water containing 0.05% PVA and DZNep (10 mg/mL in nanopure H_2_O) under sonication using conditions as mentioned above. Organic solvent was removed by washing three times using an Amicon filtration membrane with a 100 kDa cut-off. The NPs were resuspended in nanopure water and stored at 4°C until further use. DLS measurements were carried to determine size, polydispersity index (PDI), and zeta potential of the NPs. NPs were characterized using TEM as mentioned above.

### Synthesis of Controlled Release Gem-DZNep-DSPE-PEG-OH-NPs

Gemcitabine (10 mg/mL in nanopure H_2_O) was emulsified with PLGA-b-PEG-OH (5 mg in CH_2_Cl_2_) using probe sonication for one minute (Amplitude: 40% of 600 W power, Pulse: 1 sec on, 1 sec off). The primary emulsion was emulsified again by adding the water-in-oil emulsion to 2 mL of nanopure water containing 0.5% PVA and sonicated using the conditions mentioned above. This emulsion was further emulsified using 8 mL of nanopure water containing 0.05% PVA, 0.1% DSPE-PEG-COOH, and DZNep (10 mg/mL in nanopure H_2_O) under sonication using the same conditions as mentioned above. Organic solvent was removed by washing three times using an Amicon filtration membrane with a 100 kDa cut-off. The NPs were resuspended in nanopure water and stored at 4°C until further use. DLS measurements were carried to determine size, polydispersity index (PDI), and zeta potential of the NPs. NPs were characterized using TEM as mentioned above.

### Loading and Encapsulation Efficiency of Gemcitabine and DZNep in NPs

Gemcitabine and DZNep loading and encapsulation efficiency (EE) were determined by dissolving the polymeric core in 0.1 M NaOH and quantifying the amount of therapeutic in the NPs using HPLC (Mobile phase: acetonitrile with 20% H_2_O and 0.1% trifluoroacetic acid, Wavelength used: 270 nm.

### Release Kinetic Studies of DZNep/Gemcitabine NPs

NPs were placed in Slide-A-Lyzer® MINI dialysis units with a MW cutoff of 10,000 g/mol. Samples were dialyzed against 1×PBS (6.0 L) in a constant temperature shaker at 37°C and 35 RPM. At various pre-determined time points, samples were removed and the gemcitabine/DZNep content was determined by dissolving the polymeric core in 0.1 mMol NaOH and quantifying by HPLC analysis (Mobile phase: acetonitrile with 20% H_2_O and 0.1% trifluoroacetic acid, Wavelength used: 270 nm).

### Statistical Analyses

For instances where only two conditions were compared, the student's t test was used to identify significant differences. In cases where three or more conditions were compared, one-way ANOVA was conducted followed by Tukey’s Multiple Comparison Test. Each experiment was repeated at least three times. Unless otherwise indicated, *p*<0.05 and *p*<0.01 compared with control conditions are represented by one and two asterisks, respectively.

## Results

### DZNep Selectively Augments Gemcitabine Cytotoxicity in Cancerous but not Normal Pancreatic Cells

**Figure 1:**
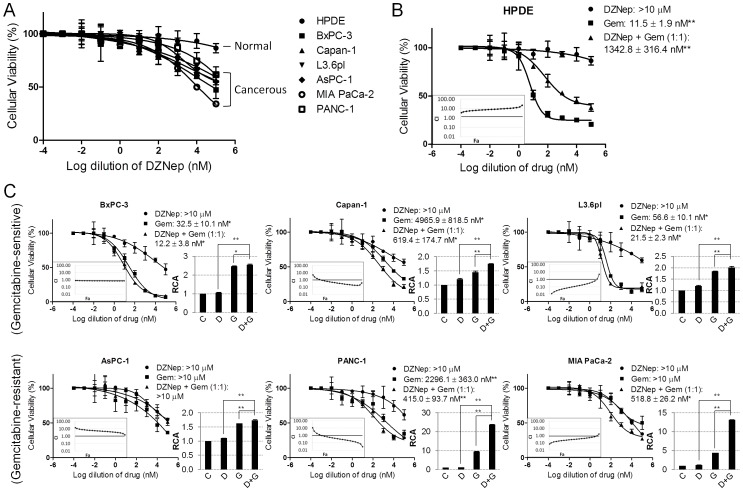
DZNep and gemcitabine sensitivity, singly or in combination, and interactions within a panel of pancreatic cell lines. A. All cancerous cell lines excluding the normal HPDE are DZNep-responsive and reduced cellular viability. B. DZNep and gemcitabine displayed antagonistic effects in HPDE. C. DZNep and gemcitabine displayed additive or synergistic effects in many of the cancerous pancreatic cell lines. Twenty-four hours after 3×10^3^ cells/well were seeded in a 96-well plate, cells were treated with either DZNep, gemcitabine, or a combination of both at an equimolar ratio for 72 h. Cellular viability was measured using an MTT assay. Cytotoxic IC_50_ values are indicated. Significances between gemcitabine and DZNep as well as DZNep+Gemcitabine and DZNep were identified using one-way ANOVA followed by Tukey’s post-hoc test. Combination index (CI) plots (insets) show the interactions between the two drugs. CI>1, antagonism; CI = 1, additivity; CI<1, synergism. Bar graphs to the right indicate the relative caspase-3 activity (RCA) of each treatment as measured by fluorescence intensity. Values were background-subtracted and are presented as fold-change from the control. Significance between a single drug versus the drug combination was identified via one-way ANOVA followed by Tukey’s post-hoc analysis. Cells were treated with 1 µM DZNep, 100 nM gemcitabine, or both. *Bars*, SD. *n* = 3. *p<0.05, **p<0.01.

We began by studying the cytotoxicity of DZNep on normal and cancerous pancreatic cell lines. Treatment with increasing concentrations of DZNep (0.1 nM-100 µM) showed a significant reduction in cellular viability in all six pancreatic cancer cell lines tested (i.e., BxPC-3, Capan-1, L3.6pl, AsPC-1, MIA PaCa-2, PANC-1) (Fig. 1A). Cytotoxicity was observed starting at approximately 0.5–1 µM DZNep, depending on the cell line, and increased gradually with higher concentrations thereafter. Cytotoxicity did not begin until ∼48 h of treatment (data not shown). Maximal reduction in cell viability (∼50% reduction with 10 µM DZNep for 72 h) was observed in poorly-differentiated MIA PaCa-2 which is only marginally gemcitabine-sensitive (Fig. 1A). In subconfluent cells, the effect of DZNep on MIA PaCa-2 was almost similar to that of gemcitabine (Fig. 1C). DZNep showed much lower cytotoxicity in well-differentiated, gemcitabine-sensitive pancreatic cancer cell lines (i.e., BxPC-3, Capan-1, L3.6pl) when compared with gemcitabine (Fig. 1C). Of note, no significant changes were observed in the proliferation and cellular viability of normal human pancreatic ductal epithelial (HPDE) cells (up to 10 µM DZNep for 72 h) which are highly gemcitabine-sensitive, as well as two other normal cell lines (293T (human embryonic kidney) and MCF-10A (human breast epithelial)) (Figs. 1A-B, S1). These results demonstrate that DZNep selectively imparts cytotoxic effects to (poorly-differentiated) pancreatic cancer cells without significant impairment of proliferation in normal pancreatic epithelial cells.

Encouraged by the potential ability of DZNep to compensate for nucleoside analog refractoriness in pancreatic cancer, we co-treated cells with DZNep and gemcitabine at equimolar ratios and compared the cellular viabilities with those obtained when cells were treated with either DZNep or gemcitabine alone. Interestingly, the co-treatment showed significantly higher reductions in cellular viabilities of most of the gemcitabine-sensitive and -insensitive cancerous cells than when the compounds were used as stand-alone agents (Fig. 1C). Conversely, the co-treatment reduced cytotoxicity (>2-fold at 100 nM concentration) in HPDE (Fig. 1B), suggesting a cytoprotective role of DZNep only on normal cells.

We estimated the combination index (CI) plots [26] to identify the type of interaction between DZNep and gemcitabine. These estimations identified a synergistic or additive interaction between DZNep and gemcitabine (100 nM-10 µM) in all pancreatic cancer cell lines with the only exception of AsPC-1, as well as a stark antagonistic interaction in normal HPDE (Fig. 1C). Co-treatment of DZNep (1 nM-10 µM) with differing gemcitabine concentrations (1–100 nM) showed that DZNep potentiates cytotoxicity in a gemcitabine dose-dependent fashion in marginally gemcitabine-sensitive MIA PaCa-2, whereas such a potentiation occurred in a largely gemcitabine dose-independent fashion in highly gemcitabine-sensitive Capan-1 cells (Fig. S2). HPDE remained cytotoxic to gemcitabine, confirming antagonism between the two compounds (Fig. S2). Further interaction analysis with various ratios of gemcitabine:DZNep (1∶1, 1∶4, 4∶1, 1∶10, 10∶1) identified a maximally synergistic and cytotoxic response in MIA PaCa-2 using the 1∶10 ratio (Fig. S3). Finally, in order to understand the mechanism of reduction in cellular viability, we conducted caspase-3 based apoptosis assays. These results (Fig. 1C) identified a significant induction of apoptosis as a major contributing mechanism for DZNep-induced increase in gemcitabine cytotoxicity (Fig. 1C).

### Nucleoside Transporters Facilitate Cellular Entry of DZNep

**Figure 2:**
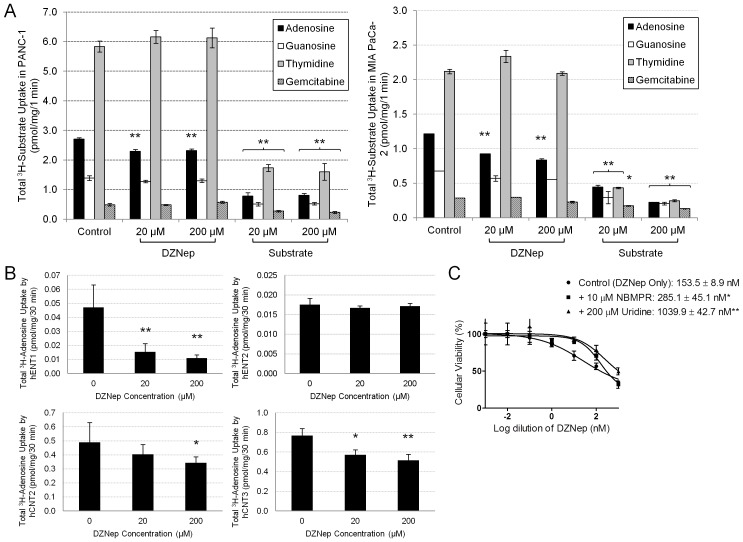
DZNep partially competes with the uptake of purine nucleosides by hENT1 and hCNT3. A. DZNep hindered the uptake of radiolabeled purine nucleosides in PANC-1 and MIA PaCa-2. Twenty-four hours after 5×10^4^ cells/well were seeded in a 24-well plate, cells were allowed to uptake the indicated radiolabeled nucleoside in the presence of DZNep or its respective unlabeled nucleoside. B. Inhibition of adenosine transport in *Xenopus* oocytes with DZNep. C. Pharmacological inhibition of hENT1 and excess uridine decreased the cytotoxicity of DZNep in MIA PaCa-2. Twenty-four hours after 3×10^3^ cells/well were seeded in a 96-well plate, cells were treated with increasing concentrations of DZNep in the presence of DMSO (control), 10 µM NBMPR, or 200 µM uridine. Cellular viability was measured using an MTT assay. IC_50_ values are indicated. Significant differences between the control and each treatment were determined using the Student’s t test. *Bars*, SD. *n* = 3. *p<0.05, **p<0.01.

Since DZNep is a hydrophilic nucleoside analog with a log P of −1.38 [5], we hypothesized that its entry into cells would depend on the expression of nucleoside transporters, and therefore, the lack of sufficient carrier expression would limit its cellular activity. To test this, we performed a competitive assay by assessing the level of inhibition in the cellular transport of nucleosides with DZNep. We chose the PANC-1 and MIA PaCa-2 cell lines for these studies because they have been reported to express multiple nucleoside transporters [33]. Transport analysis identified a significant inhibition of ^3^H-adenosine and ^3^H-guanosine (purines) transport but not ^3^H-thymidine (pyrimidine) (Fig. 2A). Gemcitabine transport in both cell types was only inhibited at a higher concentration (200 µM) (Fig. 2A). These data suggest that DZNep cellular influx is facilitated by purine, but not pyrimidine, nucleoside transporters.

To further characterize the identity of the purine nucleoside transporters mediating DZNep influx, we examined the ability of DZNep to inhibit ^3^H-adenosine transport in *Xenopus* oocytes expressing individual transporters. A significant inhibition of hENT1- and hCNT3-mediated ^3^H-adenosine transport was observed, whereas a significant inhibition of hCNT2-mediated ^3^H-adenosine transport was noticed only at the higher (200 µM) concentration (Fig. 2B). No significant reduction in adenosine transport by DZNep was observed in hENT2-expressing oocytes.

Next, to study the impact of hENT1 and hCNT3 on DZNep-mediated cytotoxicity in pancreatic cancer cells, we examined the proliferation of MIA PaCa-2 in the presence of a pharmacological inhibitor of hENT1 (10 nM NBMPR) or excess uridine (200 µM) which competitively inhibits all ENTs and CNTs. Our results show that both conditions can significantly reduce DZNep cytotoxicity of MIA PaCa-2, as judged by increases in cytotoxic IC_50_ estimates (2–8-fold) (Fig. 2C).

### Acyl Derivatization of DZNep Augments Sensitization of Pancreatic Cells to Gemcitabine

**Figure 3:**
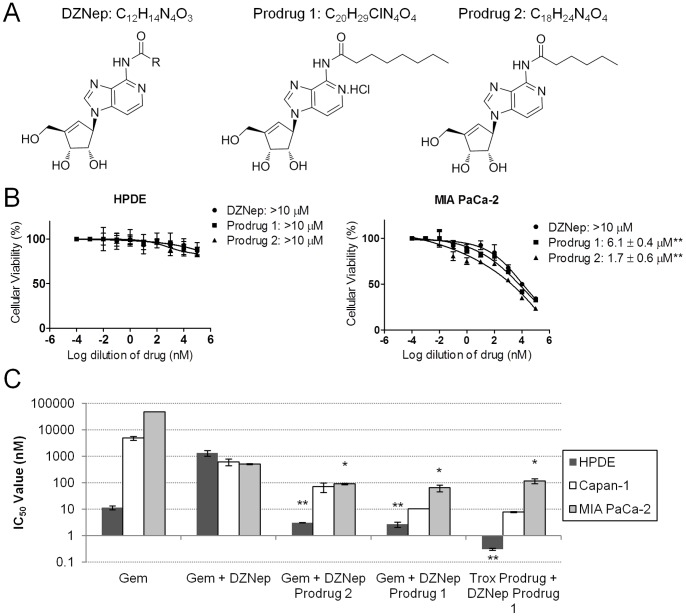
Acyl modifications of DZNep further enhance cytotoxicity. A. The chemical structures of DZNep and its two acyl prodrugs (Prodrug 1: C_20_H_29_ClN_4_O_4_, and Prodrug 2: C_18_H_24_N_4_O_4_). B. Cytotoxicity of DZNep versus its prodrugs in HPDE and MIA PaCa-2. IC_50_ values are designated in each legend. Significance between each prodrug and DZNep was identified using the Student’s t test. C. Average IC_50_ values of the various drug combinations in HPDE, Capan-1, and MIA PaCa-2. Twenty-four hours after 3×10^3^ cells/well were seeded in a 96-well plate, cells were treated for 72 h. Cellular viabilities were measured using MTT assays. IC_50_ values are plotted. Significance of each prodrug combination was compared with Gem+DZNep using one-way ANOVA followed by Tukey’s post-hoc test. *Bars*, SD. *n* = 3. *p<0.05, **p<0.01.

Since DZNep at least partially utilizes transporters for exerting its fullest potential, it is likely that transporter-deficient patients may respond poorly to DZNep. To circumvent this issue, we generated polar acyl derivatives of DZNep (Fig. 3A) using a synthetic procedure described in *Materials and Methods*. We substituted DZNep at the N_6_-NH_2_ group with acyl side chains to generate two lipophilic prodrugs (Prodrug 1: C_20_H_29_ClN_4_O_4_; Prodrug 2: C_18_H_24_N_4_O_4_). The synthesized prodrugs were evaluated for their anti-proliferative activities, both alone and in combination with gemcitabine, in a normal (HPDE), gemcitabine-sensitive (Capan-1), and gemcitabine-resistant (MIA PaCa-2) cell line. Both prodrugs showed higher cytotoxicity than the parent DZNep in MIA PaCa-2 (Fig. 3B). Further, the levels of cytotoxicity produced by both of the prodrugs were unaffected in the presence of 10 nM NBMPR or 100 µM uridine since the estimated IC_50_ values were not significantly different with the treatments. In normal HPDE, cytotoxicity of the prodrugs did not differ from that of DZNep (>10 µM) (Fig. 3B). When cells were treated with both gemcitabine and a DZNep prodrug at the previously calibrated 1∶10 ratio, both prodrugs showed a distinct potentiation of gemcitabine cytotoxicity in the cancerous Capan-1 and MIA PaCa-2 (IC_50_ reduction of ≥12-fold in Capan-1 and ≥7-fold in MIA PaCa-2) (Fig. 3C). Despite anticipated inertness, both prodrug combinations with gemcitabine increased cytotoxicity in HPDE (Fig. 3C). Overall, cytotoxicity increased from gemcitabine as a single agent, to a combination with DZNep, to a combination with DZNep prodrugs (Fig. 3C). Since gemcitabine is well-known to rely heavily on nucleoside transporters for cellular activity, we examined the effects of an acyl derivative (C_24_H_41_N_3_O_5_) of a less hydrophilic nucleoside analog, troxacitabine (log P of −0.66) [29], [30], in combination with DZNep Prodrug 1 on the same cell lines. Cytotoxicity was further enhanced with the use of the troxacitabine prodrug in combination with DZNep Prodrug 1 (Fig. 3C). While apoptosis is identified as a contributing mechanism for DZNep cytotoxicity, other possible mechanisms including autophagy or senescence may be responsible for the large reduction in cellular viability with multiple drugs. Taken together, these results indicate that acyl derivatives of one or both nucleosides can substantially potentiate the anti-proliferative effects of pancreatic cancer cells by overcoming dependence on cellular transport barriers. However, these results also indicate that the prodrugs may compromise the selective anti-proliferative effects seen in parent DZNep since cytotoxicity also increased in HPDE.

### DZNep Rapidly and Reversibly Decreases H3K27 and H4K20, but Increases H3K9, Trimethylations in Pancreatic Cancer Cells

**Figure 4:**
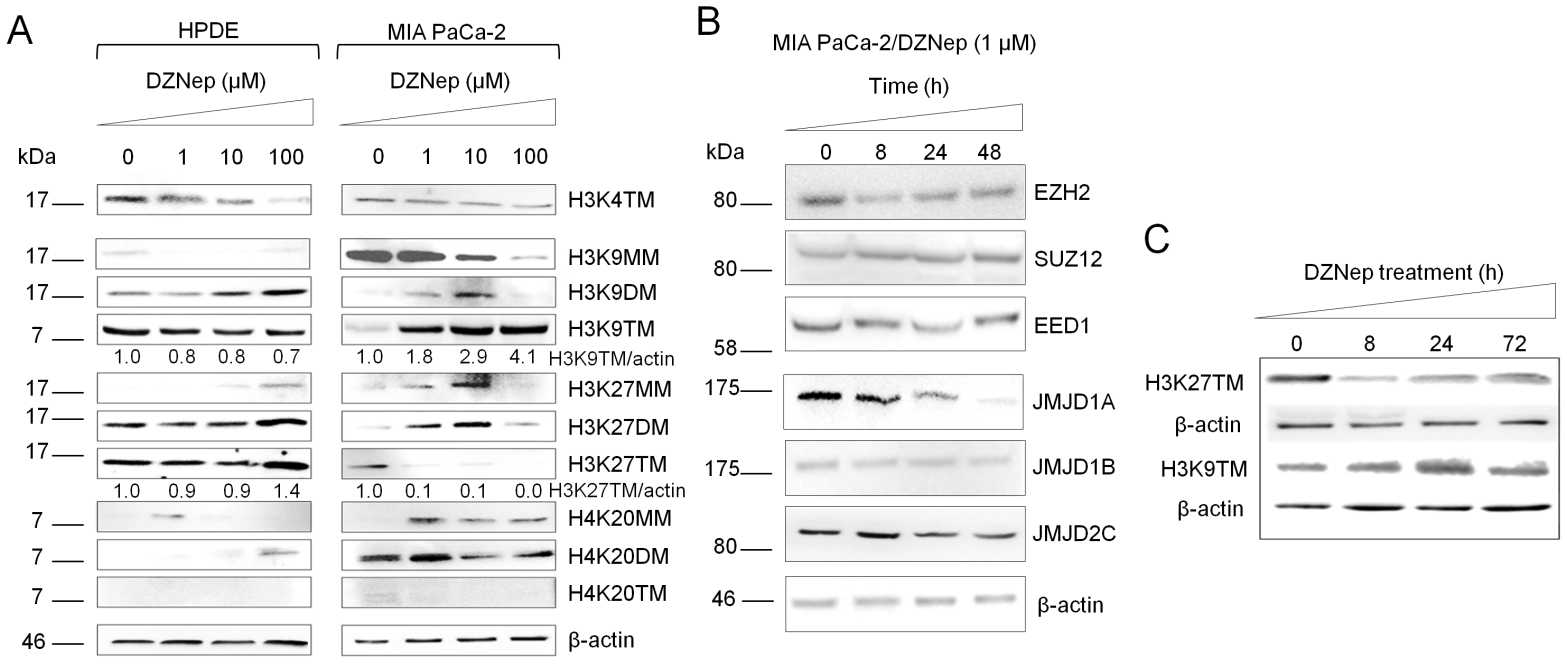
DZNep alters histone lysine methylation and methyltransferase and demethylase expressions in pancreatic cancer. A. Changes in methylation levels of H3K4, H3K9, H3K27, and H4K20 in HPDE and MIA PaCa-2 treated with DZNep (0–100 µM). Cells were treated with DZNep for 24 h, and whole cell lysates (50 µg) were subjected to Western blotting analysis. β-actin, the internal loading control, is shown with a representative blot. B. Western blotting analysis of histone lysine methyltransferases and demethylases in MIA PaCa-2 treated with DZNep (1 µM) for up to 48 h. C. Histone methylation dynamics in MIA PaCa-2 treated with DZNep (1 µM) for up to 72 h.

To investigate whether DZNep influences chemosensitivity in pancreatic cancer by affecting histone methylation-dependent chromatin states, we profiled methylation marks in key lysine residues in histones H3 and H4. Western blotting analyses indicated that in untreated conditions, H3K9 was predominantly monomethylated, H3K27 was trimethylated, and H4K20 was dimethylated in MIA PaCa-2 (Fig. 4A). Treatment with increasing concentrations of DZNep (1–100 µM) for 24 h clearly reversed at least two of these marks viz., increased H3K9me3 and decreased H3K27me3 (Fig. 4A). Further, the DZNep-induced increase in H3K9me3 was accompanied by a decrease in H3K9me1/2 forms; conversely, the DZNep-induced decrease in H3K27me3 was accompanied by an increase in H3K27me1/2 forms (Fig. 4A). DZNep treatment in MIA PaCa-2 also slightly decreased H4K20, but not H3K4, trimethylation states. Further time course analyses of the two distinct changes in the histone trimethylation states (i.e., H3K27 and H3K9) identified decreases in H3K27me3 to occur as early as 8 h of DZNep treatment, whereas the increase in H3K9me3 peaked only after 24 h of treatment (Fig. 4C). Unlike MIA PaCa-2, DZNep treatment in HPDE cells produced no obvious changes in methylation states of any of the histone lysines tested, suggesting that DZNep-induced chromatin alterations were selective to cancerous pancreatic cells (Fig. 4A).

To test the mechanisms by which these methylation changes occurred, we profiled the expressions of putative histone lysine methyltransferases and demethylases implicated in these processes. Specifically, we tested changes in the levels of the PRC2 subunits (EZH2, SUZ12, and EED) and JMJD histone demethylases (JMJD1A, JMJD1B, and JMJD2C) which have been reported to selectively target the methylation and demethylation reactions in H3K27 and H3K9 residues, respectively. Western blotting analyses of whole cell lysates prepared from DZNep-treated MIA PaCa-2 showed a moderate decrease in EZH2 histone methyltransferase and significant decreases in JMJD1A and JMJD2C histone demethylases correlating to changes observed in H3K27 and H3K9 methylations states, respectively (Fig. 4B). No significant changes were observed in SUZ12, EED, and other JMJD subunits upon DZNep treatment (Fig. 4B).

### Short DZNep Priming Prior to Gemcitabine Treatment Shows Superior Chemosensitizing Effects Compared with Simultaneous Exposure of Both Drugs: Inhibition of H3K27me3 as a Putative Mark for DZNep Chemosensitization

**Figure 5:**
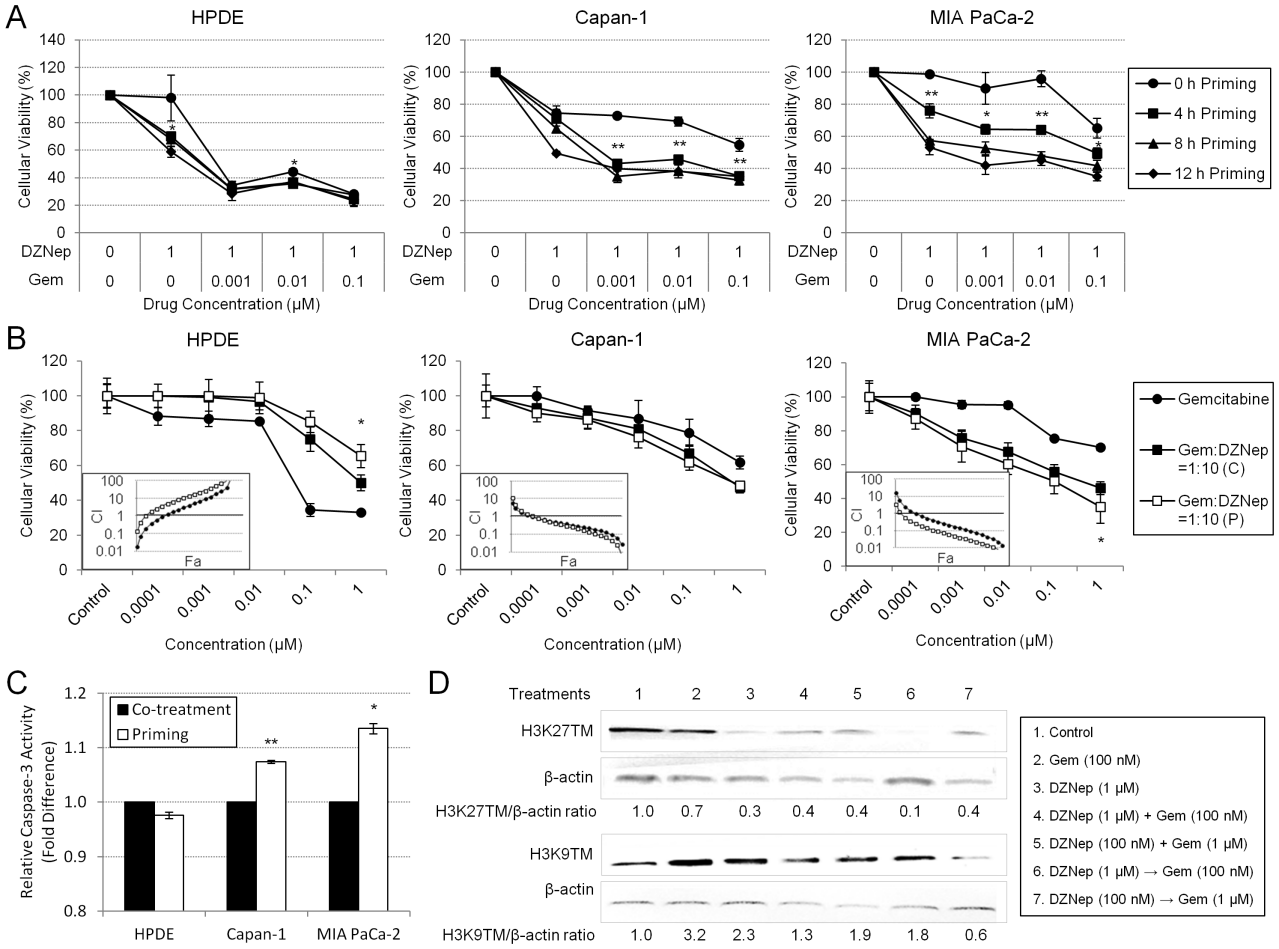
Short priming of DZNep demonstrated superior cytotoxicity and synergy with gemcitabine than co-exposure of the two drugs. A. Short exposure with DZNep for 4–8 h produced maximal cytotoxic effects. Cells were exposed with DZNep at 1 µM for varying time intervals followed by increasing concentrations of gemcitabine (0–0.1 µM). Significance between 0 and 4 h is indicated. B. Superior cytotoxicity and synergism between gemcitabine and DZNep were observed when cells were primed with DZNep, as opposed to cotreatment with gemcitabine. Representative growth inhibition curves are shown. Twenty-four hours after 3×10^3^ cells/well were seeded in a 96-well plate, cells were exposed to gemcitabine and DZNep concentrations at a 1∶10 ratio either as a co-treatment for 72 h (C) or a primed treatment (with DZNep for 8 h followed by gemcitabine for 72 h) (P). Cellular viabilities were measured using MTT assays. Significance between co-treatment and priming is indicated. Combination index (CI) plots (insets) show the interactions between the two drugs. CI>1, antagonism; CI = 1, additivity; CI<1, synergism. *Bars*, SD. *n* = 3. *p<0.05, **p<0.01. C. Apoptosis levels were significantly greater in Capan-1 and MIA PaCa-2 cells with priming compared with co-treatment, while apoptosis levels in HPDE decreased with priming. Cells were either co-treated with 10 µM DZNep and 1 µM gemcitabine or primed with 10 µM DZNep for 8 h followed by 1 µM gemcitabine. Fluorescence values were background-subtracted and are indicated as fold-change from co-treatment to priming. Significant differences between co-treatment and priming were identified using the Student’s t test. *Bars*, SD. *n* = 3. *p<0.05, **p<0.01. D. Maximal reduction in H3K27 trimethylation was seen with priming schedules at 1∶10 DZNep:gemcitabine. MIA PaCa-2 was treated with vehicle, gemcitabine for 72 h, DZNep for 8 h, DZNep and gemcitabine for 72 h, or DZNep for 8 h followed by gemcitabine for 72 h. 100 µg of whole cell lysates were subjected to Western blotting analysis. Blots were stripped and re-probed for β-actin, the internal loading control. Densitometry ratios are indicated.

To test whether the temporal differences in various lysine trimethylation changes correspond with changes in gemcitabine chemosensitization, we next performed gemcitabine cytotoxicity analyses after DZNep priming of Capan-1 and MIA PaCa-2 for various time periods (4, 8, and 12 h). Consistent with early and rapid inhibition of H3K27me3, maximal gemcitabine chemosensitization was also observed in dosing schedules where the cells were primed with DZNep for only a short 4–8 h period prior to gemcitabine treatment, and the changes did not significantly increase any further with a later priming time (12 h) (Fig. 5A). Such increases in synergistic drug responses with short DZNep priming were also greater than that observed when DZNep was co-treated with gemcitabine for the entire 72 h (Fig. 5B). A decrease in cellular viability in MIA PaCa-2 was evident throughout, but only statistically significant at the highest concentration. For Capan-1, no statistical differences were observed; however, synergism was distinctly increased with priming. Intriguingly, short priming of DZNep for 8 h augmented its antagonistic interaction with gemcitabine in HPDE causing a further reduction in the extent of cytotoxicity (Fig. 5B). Statistically significant increases in caspase-3 levels further support apoptosis as a major contributing factor to the observed reduced cellular viability in pancreatic cancer cells (Fig. 5C). Taken together, these data suggest that short DZNep priming rather than continuous co-treatment may chemosensitize pancreatic cancer cells to gemcitabine both in an effective and a selective manner.

Finally, we tested the H3K27 and H3K9 trimethylation statuses during the entire period of treatment with both DZNep and/or gemcitabine at varying schedules (short priming or co-exposure) and ratios (1∶10 or 10∶1). Maximal reductions in H3K27me3 were noticed with the DZNep short priming schedule followed by gemcitabine treatment at a 10∶1 ratio (Fig. 5D). Although H3K9me3 was increased in the aforementioned schedule, it was essentially less differentiable from the co-exposure schedule as well as when the DZNep:gemcitabine ratios were different. Furthermore, the highest increase in H3K9me3 was noted with the gemcitabine-alone schedule for the entire 72 h period, where cytotoxicity observed was the minimal among all schedules tested (Fig. 5D). These data further provide evidence that the loss of H3K27me3, and not increase in H3K9me3, is the closest predictor of DZNep potentiation of gemcitabine chemosensitivity in pancreatic cancer cells.

### An Engineered Nanoparticle for Spatiotemporal Release of DZNep and Gemcitabine Reduces DZNep Dose while Bringing Maximal Chemosensitivity

**Figure 6:**
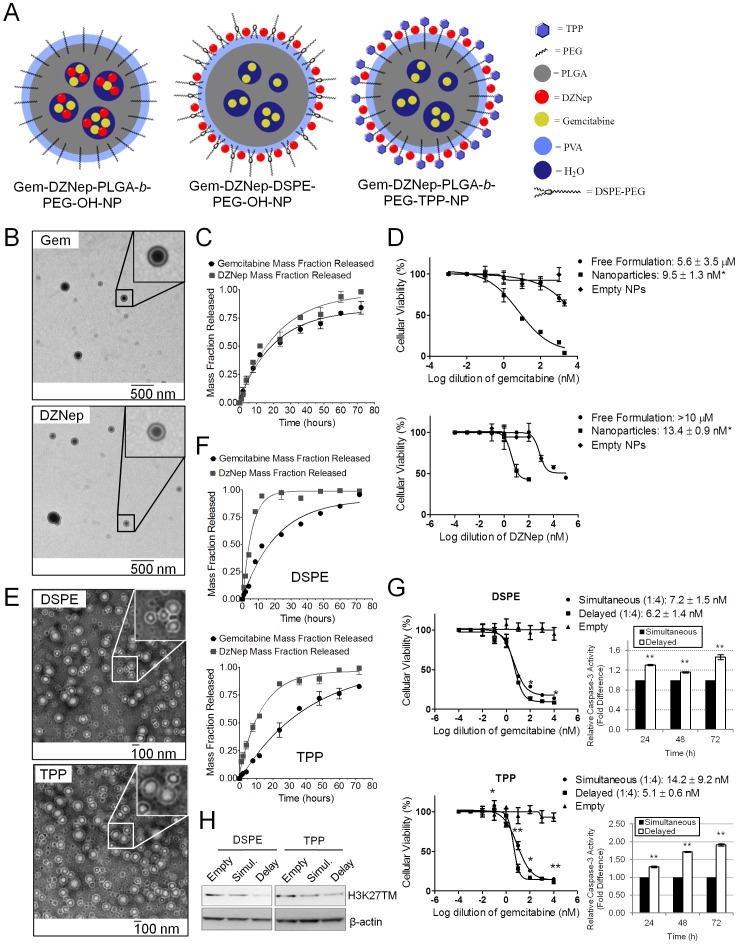
Spatiotemporal release of DZNep and gemcitabine using engineered nanoparticles reduced drug dose while potentiating chemosensitivity. A. Spatial distribution of DZNep and gemcitabine within NPs. Co-encapsulating double-emulsion formulations were created using PLGA-*b*-PEG-OH (*left*), DSPE-PEG-OH (*middle*), and PLGA-*b*-PEG-TPP (*right*). B and E. TEM illustrates the inner core and outer shell of all the double-emulsion NPs created. Insets show the NPs at higher magnification. *Bars*, SD. *n* = 3. C. Release kinetics indicates the similarity between DZNep and gemcitabine release using the PLGA-*b*-PEG-OH formulation. D. Gemcitabine (*top*) and DZNep (*bottom*) in individual PLGA-*b*-PEG-OH formulations distinctly increased cytotoxicity in MIA PaCa-2. Significance between nanoparticles and free formulation is shown. F. HPLC analyses demonstrate the rapid and sequential release of DZNep compared with gemcitabine in both DSPE (*top*) and TPP (*bottom*) formulations.G. Both engineered DSPE (*top*) and TPP (*bottom*) delayed-release NPs increased the cytotoxicity of MIA PaCa-2 even further compared with PLGA-*b*-PEG-OH NPs. Twenty-four hours after 5×10^3^ cells/well were seeded in a 96-well plate, cells were treated for 72 h. Cellular viabilities were measured using MTT assays. Cytotoxic IC_50_ values are indicated. Significance between simultaneous and delayed NPs using the Student’s t test is shown. Bar graphs to the right indicate the relative levels of caspase-3 activity as measured by fluorescence intensity. Values were background-subtracted and are indicated as fold-change from simultaneous NPs to delayed NPs. *Bars*, SD. *n* = 3. *p<0.05, **p<0.01. H. H3K27 trimethylation decreases with simultaneous and delayed-release NPs. MIA PaCa-2 was treated with empty, simultaneous, or delayed NPs as above and total cell lysates were analyzed for H3K27 trimethylation using Western Blotting. A clear reduction in H3K27 trimethylation was noticed in both simultaneous and delayed NPs, with DSPE (*left*) and TPP (*right*) delayed NPs producing a slightly greater reduction compared with simultaneous NPs.

To enhance tumor cell delivery of the drugs, we encapsulated DZNep and gemcitabine individually into biodegradable poly(lactide-*co*-glycolide)-*b*-polyethyleneglycol (PLGA-*b*-PEG) nanoparticle (NP) formulations (Figs. 6A, S4). Transmission electron microscopy (TEM) studies showed a uniform size distribution of both DZNep and gemcitabine NPs ranging between 100–200 nM (Fig. 6B). *In vitro* release kinetics suggested a similar release profile for both drugs with almost comparable physicochemical characteristics (Fig. 6C; Table 2). Cytotoxicity analyses with drug-encapsulated NPs indicated that DZNep entrapment within NPs can produce the same level of cytotoxicity as that of free DZNep although at a much lower (∼10-fold) concentration (Fig. 6D). A similar reduction in dose requirement was also obtained with gemcitabine when used in NP formulation (Fig. 6D).

**Table 2:** Physiochemical Characterization of NPs.

Nanoparticle	Size (nm)	PDI	Zeta Potential (mV)	Loading (%)		EE (%)	
				Gemcitabine	DZNep	Gemcitabine	DZNep
Empty-PLGA-*b*-PEG-OH-NPs	172±2	0.13	−23.5±1.3	**–**	**–**	**–**	**–**
Gem-PLGA-*b*-PEG-OH-NPs	200±4	0.16	−27.6±0.7	1.4±0.1	**–**	6.6±0.2	**–**
DZNep-PLGA-*b*-PEG-OH-NPs	206±2	0.16	−29.6±0.1	**–**	1.9±0.1	–	9.1±0.6
Gem-DZNep-PLGA-*b*-PEG-OH-NPs	200±2	0.17	−24.3±4.9	1.3±0.1	1.7±0.2	6.2±0.5	8.2±0.7
Gem-DZNep-PLGA-*b*-PEG-TPP-NPs	157±1	0.20	16.4±0.9	1.2±0.2	1.6±0.5	5.5±0.8	7.7±2.4
Gem-DZNep-DSPE-PEG-NPs	167±2	0.23	−17.9±2.8	1.3±0.2	1.9±1.0	6.3±0.8	8.9±4.9

Since superior chemosensitization effects were seen when cells were initially primed with DZNep for a short period of time, we engineered two different NP formulations such that gemcitabine was encased in the core while DZNep was loaded within the outer layer (Figs. 6A, S4). The first strategy used DSPE-PEG-OH to create a lipid layer to accommodate DZNep alone, and the second used a PLGA-*b*-PEG-TPP polymer to keep the two drugs separated by a cationic charge (Fig. 6A, S4). TEM studies once again showed a uniform size distribution of both NP formulations (Fig. 6E). However, comparison of gemcitabine and DZNep release kinetics showed a much more rapid release of DZNep (∼80% within 8 h) than gemcitabine in the former and a slightly greater release of DZNep (∼50% within 8 h) than gemcitabine in the latter (Fig. 6F). Treatment of MIA PaCa-2 with each controlled-release NP type showed a superior cytotoxicity response than gemcitabine and DZNep co-encapsulated NPs at the same ratios (IC_50_ reductions for DSPE formulation of 1.2–2.5-fold (p = 0.48) and TPP formulation of 1.5–4.9-fold (p = 0.12)) (Fig. 6G). In both cases, a significant induction of apoptosis consequent to inhibition of H3K27me3 was identified as a key mechanism for the observed cytotoxicity (Figs. 6G,-H).

## Discussion

Advantages of gemcitabine for pancreatic cancer therapy include its mechanistic attributes such as self-potentiation and masked chain termination [34], ability to expose cancer cell antigens to trigger immune reactions, and overall well-tolerability in patients. Nonetheless, the use of gemcitabine monotherapy for pancreatic cancer only produces modest treatment and survival responses in patients. Several studies have observed potential issues associated with the continuous usage of this treatment, including increased development of stem cell characteristics and EMT behavior, enhancing chemoresistance [35]. Furthermore, innumerable studies evaluating the potential of gemcitabine in combination drug regimens with other conventional chemotherapeutics (e.g., platins, taxols) displayed a lack of synergism or even antagonistic effects [36]. Therefore, our study attempts to increase the effectiveness of gemcitabine in pancreatic cancer by reducing its disadvantageous effects while retaining beneficial anticancer responses.

DZNep by itself exerted poor cytotoxicity in pancreatic cancer cells. Delayed and moderate results were observed with effects at the most equaling gemcitabine only in poorly-differentiated pancreatic cancer cell types. However, the drug was found to increase differentiation and E-cadherin expression in other cancer types as well as potentially reprogram gene expression [2]. Therefore, we predicted that DZNep could compensate for the refractoriness acquired from gemcitabine treatment. Although the exact mechanism of DZNep cellular action is unclear, based on its hydrophilicity (log P of −1.38) [5], we speculated that DZNep cellular entry will depend on a transport process. As noted in this report, DZNep appears to only partially utilize hENT1 and hCNT3, which is relatively moderate considering its nucleoside analog structure. This suggests that the drug may also utilize other classes of transporters capable of transporting nucleosides in order to gain entry into cells. Whether diffusion also plays a role is unclear; however, it is likely. Nevertheless, the dependence on transporters for drug uptake could partially explain a previous study which noted the limited absorption, reduced biodistribution, and rapid elimination of DZNep in mice [37]. More importantly, since earlier studies identified that patients with low nucleoside transporter expressions, especially hENT1 and hCNT3, exhibit poor treatment and survival outcomes when treated with nucleoside analogs, we synthesized acyl prodrugs of DZNep to maximize its cytotoxic response. The results support increased lipophilicity as a means to bypass transport requirements and as a preferred strategy for enhancing nucleoside analog efficacy in pancreatic cancer cells [29], [30]. Second, unlike gemcitabine, DZNep is most likely not phosphorylated into an active form. Earlier studies have reported the drug to be equally effective in cells expressing or lacking adenosine kinase [14], suggesting it unlikely that phosphorylated metabolites of DZNep act as the active units for cytotoxicity. Furthermore, the structure of DZNep does not indicate the likelihood for phosphate group addition [38]. Consistently, and as demonstrated in this study, epigenetic mechanisms such as alterations in histone methylations could aid in the mechanism of DZNep action in pancreatic cancer cells.

The use of DZNep in combination with gemcitabine was investigated in detail for potential enhancement of efficacy via advantageous interactions between the two compounds. Further, since gemcitabine has the ability to enhance chemoresistance, it was crucial to consider the pattern of dose exposure in pancreatic tumors especially to minimize clonal expansion of populations with increased stemness. In this study, a prior short exposure (i.e., priming) of DZNep was found to have pronounced overall cytotoxic effects with less drug exposure compared with conventional dosing (i.e., co-treatment). This illustrated the potential of DZNep to act as a potent chemosensitizing agent, rather than a combination cytotoxic agent, for nucleoside analogs in treating pancreatic cancer. While the avoidance of transport-based drug interactions (i.e., competition for cellular entry, especially for broadly specific nucleoside transporters (hENT1, hCNT3)) could contribute to the superiority of the priming schedule, DZNep also profoundly alters histone methylation states in cancer cells. Therefore, it is speculated that the genetic reprogramming consequent to post-translational modifications of histone lysines could trigger downstream alterations of chemosensitizing factors advantageous to gemcitabine’s mechanism of action. Consistently, DZNep effects in this study were observed as early as 4 h, correlating with the approximate time needed for the transcription of new genes. DZNep-responsive genes have been examined [16], [39]; however, their roles in chemosensitivity remain unknown, warranting further studies. Since gemcitabine is a pyrimidine (cytidine) nucleoside analog and DZNep is a purine (adenosine) nucleoside analog, it is also likely that the two could impact distinct endogenous nucleotide pools and cellular targets, allowing their functions to synergize. Conversely, our studies revealed an antagonistic interaction between DZNep and gemcitabine in HPDE. While the mechanism for a cytoprotective response in normal cells is presently unclear, the opposing action of DZNep on normal and cancerous pancreatic cancer cells presents broader clinical implications for improving chemotherapeutic efficacy in pancreatic tumors.

We found DZNep to rapidly and reversibly decrease H3K27me3 and increase H3K9me3. In addition, both events appeared to be temporally separated during DZNep treatment with the loss of H3K27me3 preceding the gain of H3K9me3. While some studies show that an increase in H3K9me3 is associated with transcriptional repression, the significance of the increase during DZNep treatment remains unclear, including whether it favors or opposes chemosensitivity. However, a study by Rogenhofer et al. identified overexpressed H3K9 methylation in benign renal tissue when compared with cancerous, as well as a correlation between H3K9 and H4K20 methylation levels with renal cell carcinoma progression [40]. Furthermore, the methylation statuses of H3K9 and H3K4 (both shown to be correlated with drug sensitivity [41], [42]) with respect to DZNep treatment may simply be governed by their mutually-exclusive properties (i.e., H3K9 demethylation as a requirement for subsequent H3K4 methylation [43]). Since both decreased H3K27 and increased H3K9 trimethylations are distinctly noted upon DZNep treatment [19], we investigated these lysine alterations with cytotoxic response. Clearly, maximal reduction of H3K27me3 was noticed in the DZNep short priming schedule followed by gemcitabine treatment, corroborating the sustained inhibition of H3K27me3, and not increase in H3K9me3, as a favorable histone lysine mark for augmented nucleoside analog chemosensitization of pancreatic cancer cells.

Our data identified inhibition of JMJD1A and JMJD2C with DZNep treatment, correlating with increased H3K9me3 and consistent with the specificity of these demethylases to H3K9. However, our investigation into methylation changes in H3K27 demonstrated only moderately inhibited EZH2 protein, since it is known that EZH2 displays highest catalytic activity for the first monomethylation of H3K27 but relatively weak capability for the subsequent di- and tri-methylations [44]. Despite the common desire for a specific EZH2 inhibitor (since DZNep is a global methylation inhibitor, not specific to EZH2) [5], [19], we believe this may actually be an advantage of the drug. One study observed defects in organ development or function in mice with the inactivation of EZH2 in adult stem cells [45], suggesting austere side effects in silencing the protein entirely. In addition, reducing both mRNA and protein levels of EZH2 with RNAi has been shown to result in different patterns of PRC2 target genes expressed as compared with the pharmacological effects of DZNep on EZH2 [16]. Since DZNep alters more than just the silencing of EZH2 (such as the hypomethylation of other genes [37]), it seems the collective consequences may be what are contributing to its preferred chemosensitizing effects.

While our studies address many of the concerns previously reviewed (i.e., increasing the hydrophilicity of DZNep and investigating its potential in combination with a conventional chemotherapeutic agent [5]), the effects of DZNep on normal adult stem and progenitor cells, remain unknown [45]. As noted by Crea et al., toxicokinetics have not yet been conducted in humans, and the drug is still very investigational for its use in cancer. Further, since some of our treatments were cytotoxic to even HPDE cells (e.g., Troxacitabine Prodrug+DZNep Prodrug), the utility of DZNep may be best increased by using a targeted method. In addition, DZNep augmentation of gemcitabine chemosensitization in pancreatic cancer cell lines occurred only at a very high (≥1–20 µM) dose range. Furthermore, earlier *in vivo* mouse pharmacokinetic studies have reported that DZNep is eliminated with a short half-life (12.8 min) and only poorly distributes into peripheral tissues. Since more favorable pharmacokinetic profiles for DZNep such as dose reduction, longer circulating half-life, and improved tumor targeting due to enhanced permeation and retention (EPR) effects could all be obtained with nanoparticle drug delivery, we further investigated the delivery of DZNep in nanoparticle formulation into pancreatic cancer cells. As demonstrated in this study, engineered NPs co-encapsulating both drugs and sequentially releasing DZNep followed by gemcitabine led to improved efficacy *in vitro*, revealing the potential of the optimized epigenetic-chemotherapeutic combination with targeted drug delivery.

In summary, we have developed *in vitro* optimization procedures for the DZNep-gemcitabine combination against human pancreatic cancer cells. While gemcitabine alone only produces modest effects in the cancer, the addition of DZNep synergizes the two drugs to enhance overall efficacy in poorly-differentiated cancer cells but not normal epithelial cells. By altering structure (DZNep acyl derivatives), dose (10∶1 DZNep to gemcitabine), exposure (4 h DZNep priming prior to 72 h gemcitabine treatment), and formulation (engineered NPs), these results demonstrate potential for the use of this epigenetic-chemotherapeutic combination approach for further studies in the treatment of pancreatic cancer.

## Supporting Information

Figure S1 **Non-cytotoxic effects of DZNep on a panel of normal cell lines.** DZNep did not significantly reduce cellular viability in 293T (human embryonic kidney cells) or MCF-10A (human breast epithelial cells) (cellular viability >50% for up to 100 µM DZNep). Twenty-four hours after 3×10^3^ cells/well were seeded in a 96-well plate, cells were treated with DZNep (0–100 µM) for 72 h. Cellular viability was measured using an MTT assay. *Bars*, SD. *n* = 3.
(TIF)

Figure S2 **Chemosensitivity of normal (HPDE), gemcitabine-sensitive (Capan-1), and gemcitabine-resistant (MIA PaCa-2) pancreatic cell lines with gemcitabine and DZNep as combination agents at various concentrations.** DZNep potentiatied gemcitabine cytotoxicity in a dose-dependent fashion in HPDE and MIA PaCa-2 but a dose-independent fashion in Capan-1. Twenty-four hours after 3×10^3^ cells/well were seeded in a 96-well plate, cells were co-treated with various concentrations of DZNep (0–10 µM) and gemcitabine (0–10 nM) for 72 h. Cellular viability was measured using an MTT assay. *Bars*, SD. *n* = 3.
(TIF)

Figure S3 **Sensitivity and interactions of various ratios of gemcitabine to DZNep in MIA PaCa-2.** The greatest reduction in cellular viability as well as maximal synergistic response in MIA PaCa-2 occurred using the 1∶10 gemcitabine:DZNep (G:D) ratio. Twenty-four hours after 3×10^3^ cells/well were seeded in a 96-well plate, cells were co-treated with various ratios of gemcitabine to DZNep (0–1 µM) for 72 h. Cellular viability was measured using an MTT assay. Cytotoxic IC_50_ values are indicated. Combination index (CI) plots (insets) show the interactions between the two drugs. CI>1, antagonism; CI = 1, additivity; CI<1, synergism. *Bars*, SD. *n* = 3.
(TIF)

Figure S4 **Construction of DZNep-gemcitabine co-encapsulated nanoparticles.** Double-emulsion formulations using PLGA-*b*-PEG-OH (*top*), DSPE-PEG-OH (*middle*), and PLGA-*b*-PEG-TPP (*bottom*). Diagrams illustrate a representation of the engineered nanoparticles with the spatial distribution of both gemcitabine and DZNep for each.
(TIF)
