# Supplementary figures and images for: Pharmacological Reversal of Histone Methylation Presensitizes Pancreatic Cancer Cells to Nucleoside Drugs: In Vitro Optimization and Novel Nanoparticle Delivery Studies
Source: PLoS One. 2013 Aug 6;8(8):e71196. doi: 10.1371/journal.pone.0071196 (PMC3735519; doi:10.1371/journal.pone.0071196)

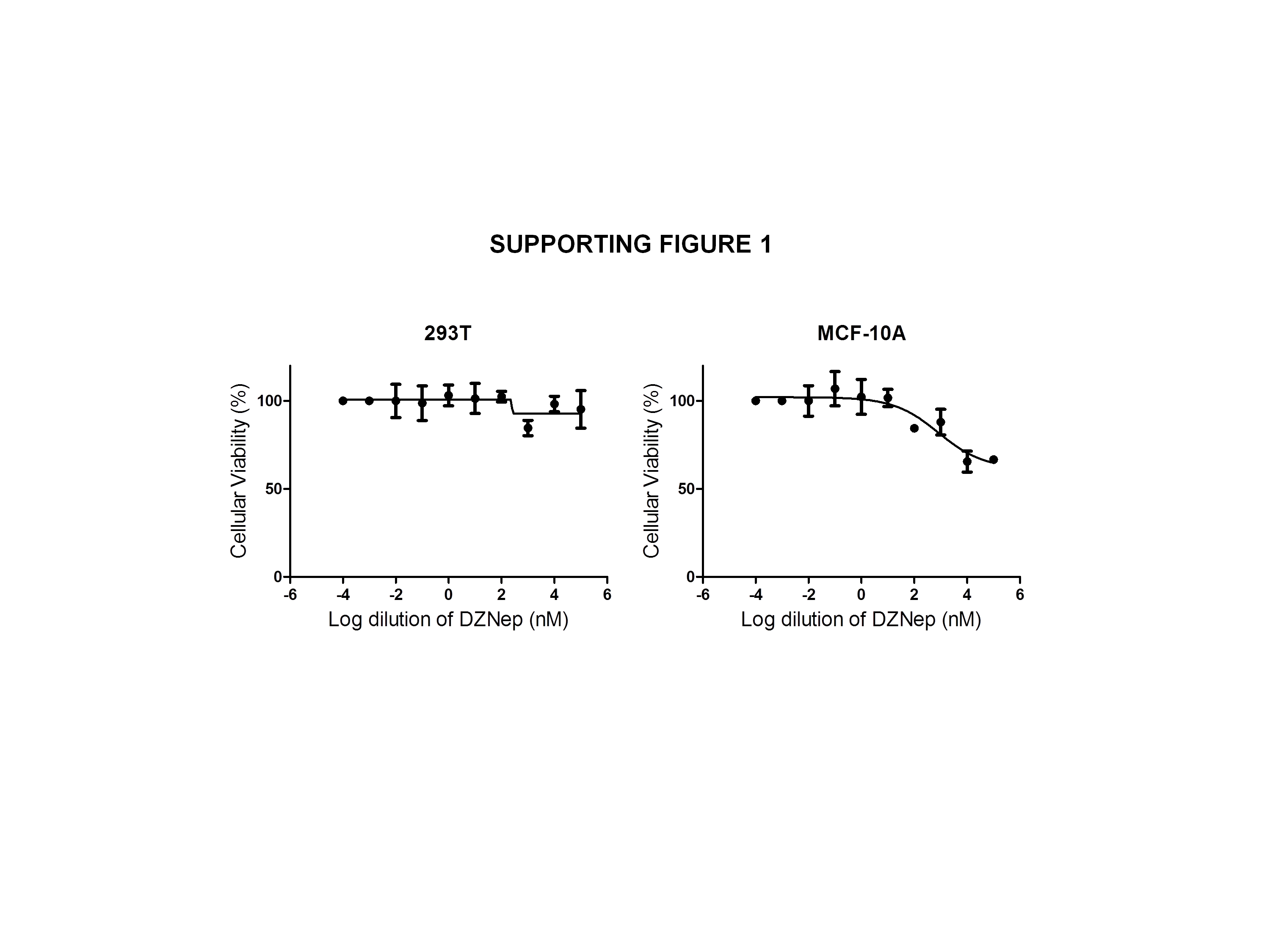

Supplement: Figure S1 — Non-cytotoxic effects of DZNep on a panel of normal cell lines. DZNep did not significantly reduce cellular viability in 293T (human embryonic kidney cells) or MCF-10A (human breast epithelial cells) (cellular viability >50% for up to 100 µM DZNep). Twenty-four hours after 3×10^3^ cells/well were seeded in a 96-well plate, cells were treated with DZNep (0–100 µM) for 72 h. Cellular viability was measured using an MTT assay. Bars, SD. n = 3. (TIF) [file pone.0071196.s001.tif]

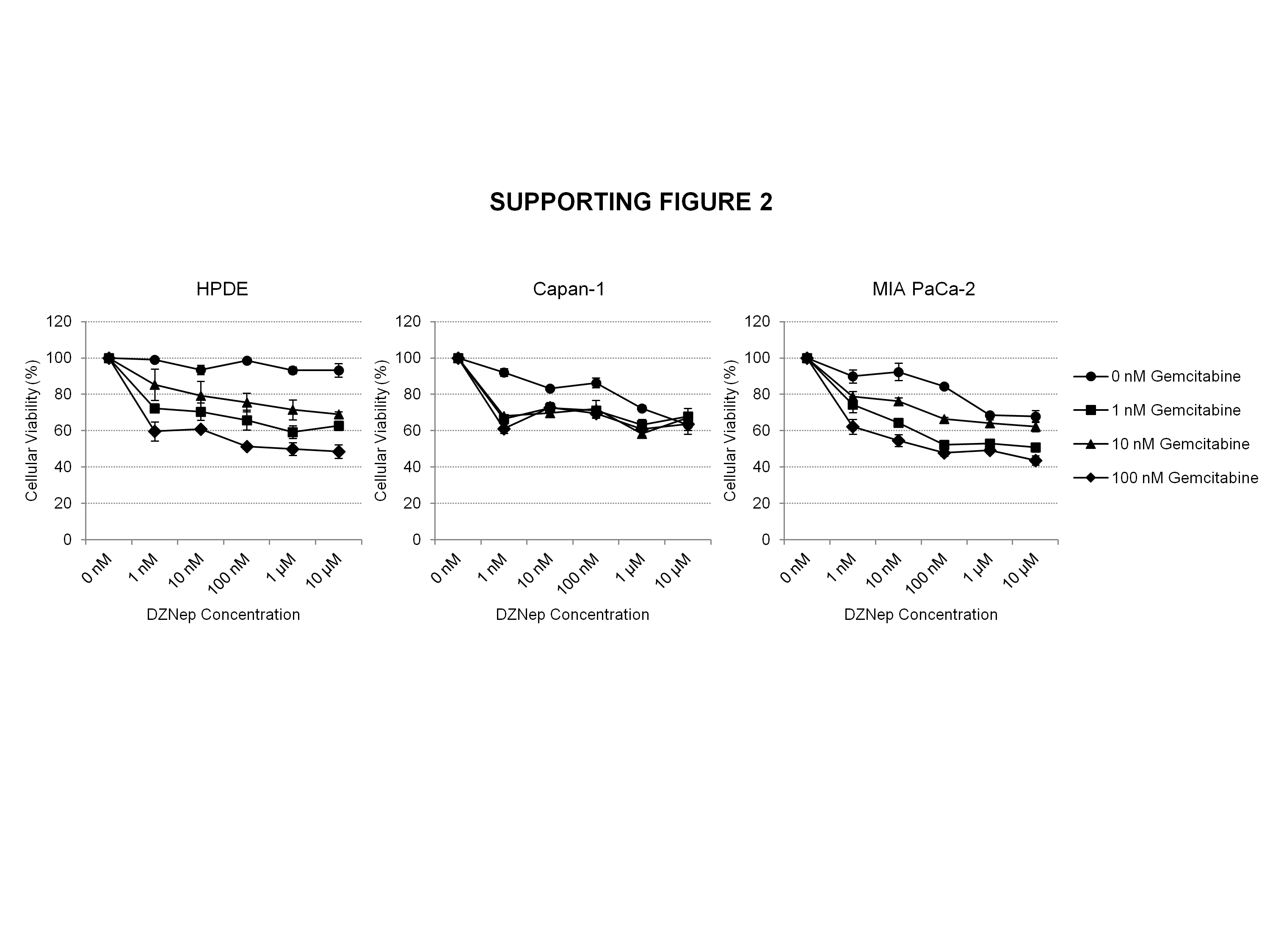

Supplement: Figure S2 — Chemosensitivity of normal (HPDE), gemcitabine-sensitive (Capan-1), and gemcitabine-resistant (MIA PaCa-2) pancreatic cell lines with gemcitabine and DZNep as combination agents at various concentrations. DZNep potentiatied gemcitabine cytotoxicity in a dose-dependent fashion in HPDE and MIA PaCa-2 but a dose-independent fashion in Capan-1. Twenty-four hours after 3×10^3^ cells/well were seeded in a 96-well plate, cells were co-treated with various concentrations of DZNep (0–10 µM) and gemcitabine (0–10 nM) for 72 h. Cellular viability was measured using an MTT assay. Bars, SD. n = 3. (TIF) [file pone.0071196.s002.tif]

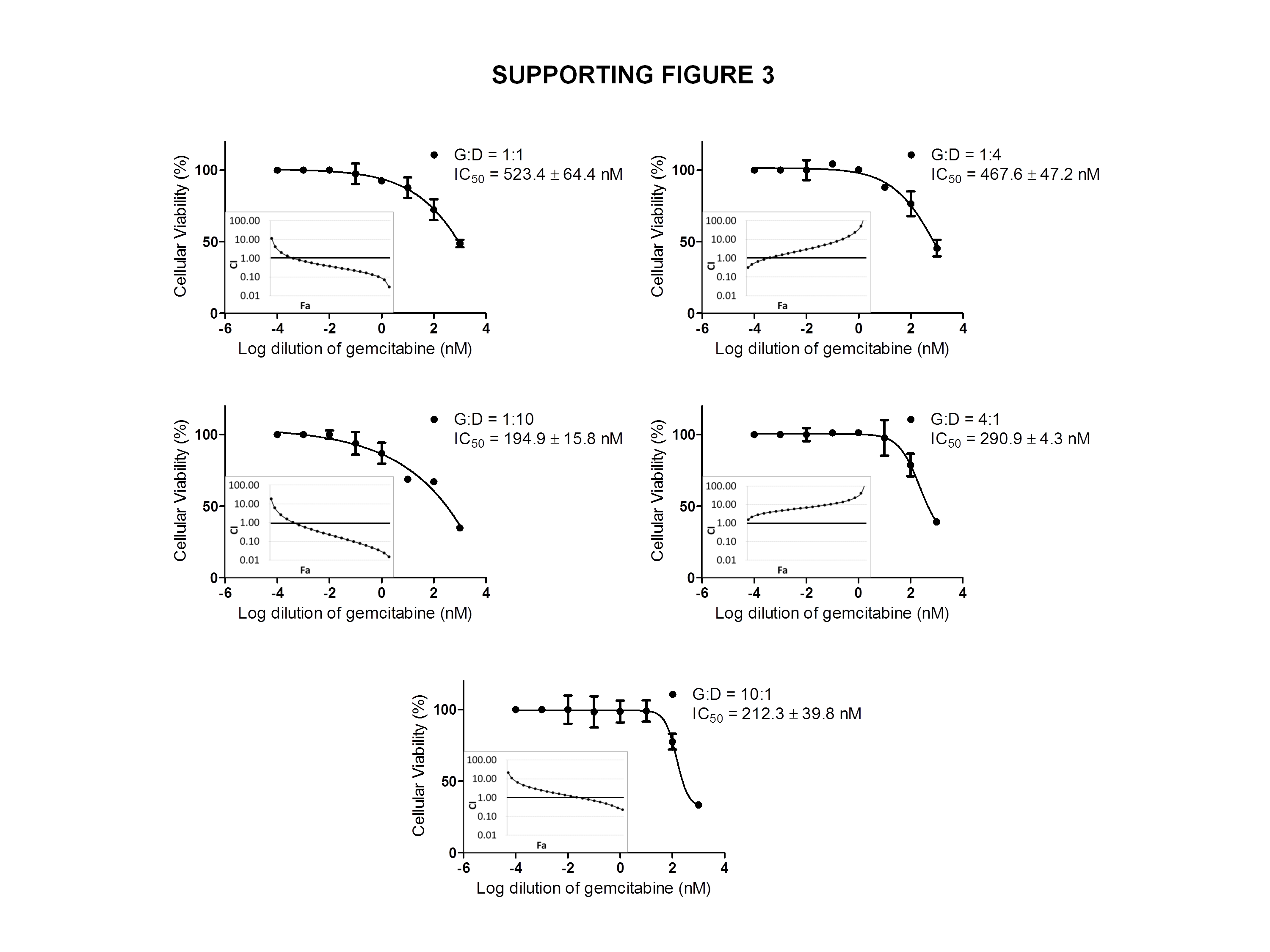

Supplement: Figure S3 — Sensitivity and interactions of various ratios of gemcitabine to DZNep in MIA PaCa-2. The greatest reduction in cellular viability as well as maximal synergistic response in MIA PaCa-2 occurred using the 1∶10 gemcitabine:DZNep (G:D) ratio. Twenty-four hours after 3×10^3^ cells/well were seeded in a 96-well plate, cells were co-treated with various ratios of gemcitabine to DZNep (0–1 µM) for 72 h. Cellular viability was measured using an MTT assay. Cytotoxic IC50 values are indicated. Combination index (CI) plots (insets) show the interactions between the two drugs. CI>1, antagonism; CI = 1, additivity; CI<1, synergism. Bars, SD. n = 3. (TIF) [file pone.0071196.s003.tif]

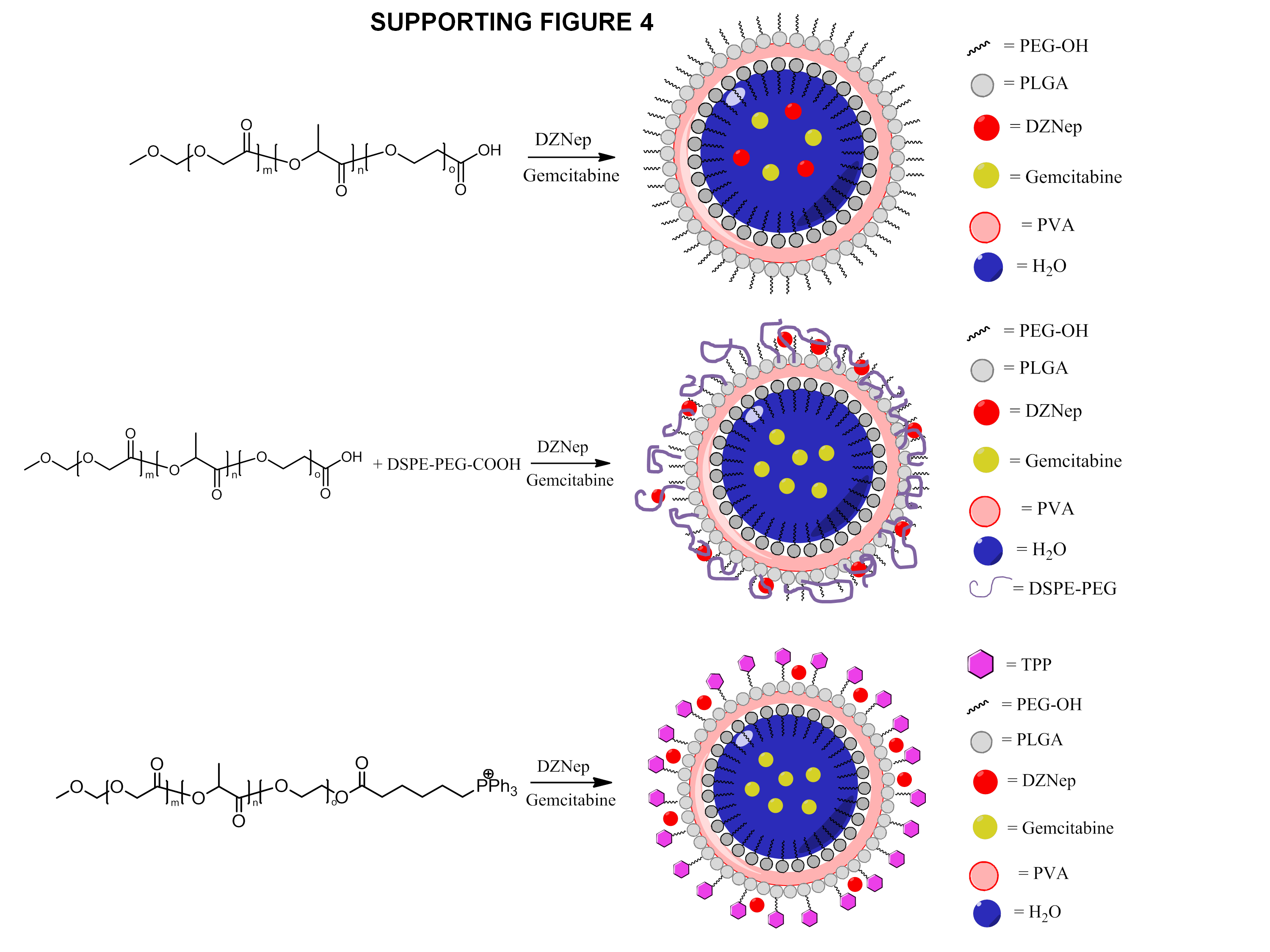

Supplement: Figure S4 — Construction of DZNep-gemcitabine co-encapsulated nanoparticles. Double-emulsion formulations using PLGA-b-PEG-OH (top), DSPE-PEG-OH (middle), and PLGA-b-PEG-TPP (bottom). Diagrams illustrate a representation of the engineered nanoparticles with the spatial distribution of both gemcitabine and DZNep for each. (TIF) [file pone.0071196.s004.tif]
